# Wild Mushrooms in Nepal: Some Potential Candidates as Antioxidant and ACE-Inhibition Sources

**DOI:** 10.1155/2014/195305

**Published:** 2014-01-28

**Authors:** Tran Hai Bang, Hiroto Suhara, Katsumi Doi, Hiroya Ishikawa, Katsuya Fukami, Gopal Prasad Parajuli, Yoshinori Katakura, Shuntaro Yamashita, Kazuo Watanabe, Mahesh Kumar Adhikari, Hira Kaji Manandhar, Ryuichiro Kondo, Kuniyoshi Shimizu

**Affiliations:** ^1^Faculty of Agriculture, Kyushu University, Fukuoka 812-8581, Japan; ^2^Department of Chemistry, Hue College of Sciences, Hue University, 77 Nguyen Hue, Hue, Vietnam; ^3^Miyazaki Prefectural Wood Utilization Research Center, Miyazaki 885-0037, Japan; ^4^International College of Arts and Sciences, Fukuoka Women's University, Fukuoka 813-8529, Japan; ^5^Material Management Center, Kyushu University, Fukuoka 812-8581, Japan; ^6^Plant Pathology Division, Nepal Agriculture Research Council, Khumaltar, Lalitpur, Nepal, P.O. Box. 3605, Kathmandu, Nepal; ^7^Graduate School of Systems Life Sciences, Kyushu University, Fukuoka 812-8581, Japan; ^8^254 Adhikari Niwas, Alka Basti, Lainchour, P.O. Box 21758, 29, Kathmandu, Nepal

## Abstract

Twenty-nine mushrooms collected in the mountainous areas of Nepal were analyzed for antioxidant activity by different methods, including Folin-Ciocalteu, ORAC, ABTS, and DPPH assays. Intracellular H_2_O_2_-scavenging activity was also performed on HaCaT cells. The results showed that phenolic compounds are the main antioxidant of the mushrooms. Among studied samples, *Inonotus andersonii*, and *Phellinus gilvus* exhibited very high antioxidant activity with the phenolic contents up to 310.8 and 258.7 mg GAE/g extracts, respectively. The H_2_O_2_-scavenging assay on cells also revealed the potential of these mushrooms in the prevention of oxidative stress. In term of ACE-inhibition, results showed that *Phlebia tremellosa* would be a novel and promising candidate for antihypertensive studies. This mushroom exhibited even higher *in vitro* ACE-inhibition activity than *Ganoderma lingzhi*, with the IC_50_ values of the two mushrooms being 32 **μ**g/mL and 2 **μ**g/mL, respectively. This is the first time biological activities of mushrooms collected in Nepal were reported. Information from this study should be a valuable reference for future studies on antioxidant and ACE-inhibitory activities of mushrooms.

## 1. Introduction

For millennia, mushrooms have been used as a part of the human diet and as medicinal sources. In term of nutrition, mushrooms are recognized as a healthy food as they are low in calories and fat but rich in proteins and dietary fiber [1, 2], while pharmacologically, the potential of medicinal mushrooms is considered enormous but mostly untapped [3]. With their wide variety of components, mushrooms—both edible and medicinal—have a broad spectrum of bioactivities [4, 5]. According to FAOSTAT data [6], the total world production of mushrooms including truffles has sharply increased from 2.0 million metric tons in 1990 to nearly 7.4 million metric tons in 2010 and the market of mushroom-derived dietary supplements is also quickly growing and is valued at more than US $15 billion today [7]. This tendency may reflect an increase in the recognition of the value of mushrooms as a healthy food and an important source of medicinal compounds.

Oxidative stress is a chronic imbalance between antioxidant ability of biological systems and production of reactive oxygen species (ROS) that is involved in many diseases including skin aging and hypertension [8–10]. UV exposure is initial step of ROS generation, causes many alterations and mutations in skin [11–13]. The skin itself has antioxidant defense system used to deactivate ROS, but when this system is overwhelmed there is a need of antioxidant supplement through food or treatment therapies [12, 14]. Abundance of studies have reported about beneficial effect of antioxidant on skin protection against ROS and were thoroughly reviewed in recent reports [12, 15–17]. Experimental and clinical studies have also indicated that hypertension occurs after a biological system is exposed to oxidative stress and increased production of ^•^O_2_^−^ and H_2_O_2_ has also been observed in salt-sensitive and angiotensin II-induced hypertension [10, 18]. Oxidative stress is both cause and effect in hypertension [19]. These findings imply that the lower level of ROS, the lower risk of hypertension and vice versa. In fact, a high intake of flavonoid-containing fruits and vegetables has been associated with a decrease in blood pressure in humans [20, 21]. Mushrooms, being neither plants nor animals, reside in their own kingdom with their own antioxidant profile and therefore have the ability to complement the benefits of antioxidants found in plant foods [22].

Besides oxidative stress-induced hypertension, a widely accepted signaling pathway of hypertension is through the angiotensin-I-converting enzyme (ACE), which plays an important role in the regulation of blood pressure. The inhibition of ACE is considered a useful therapeutic approach in the development of drugs to control hypertension. Many studies have reported on potential ACE-inhibitors from not only well-known medicinal mushrooms like *Ganoderma lucidum* and *Lentinus edodes* but also normal edible mushrooms such as *Grifola frondosa*, *Lyophyllum decastes*, and *Tricholoma giganteum* [23–27]. Many mushrooms have also shown hypotensive effects on spontaneously hypertensive rats [25, 27, 28] and humans [29]. With around 140,000 species of mushrooms estimated on earth [3], including both known and unknown species, mushrooms remain a untapped medicinal resource. It is likely that we can discover some species that are in treating other diseases as well as hypertension.

Nepal, a small country located between India and Tibet, has dramatic differences in elevation and tremendous variation in climate. With five climate zones, within a 150 km range, one can rapidly move from a typical tropic area to a permanently frozen arctic-like zone [30]. This variation has endowed the country with a diverse phytogeography, and enriched it with economically important mycoflora. Wild mushrooms are diverse and play vital roles in many local communities in Nepal [31], but surprisingly almost no published research can be found on the pharmacological potential or bioactive components of mushrooms grown in Nepal. Accordingly, the main purpose of this study was to determine the antioxidant and ACE-inhibition activities of wild mushrooms in Nepal, for many of which this is the first report on such activities.

## 2. Materials and Methods

### 2.1. Mushroom Collection and Identification

Fully matured mushrooms were collected from the forests of Kathmandu, Lalitpur, and Bhaktapur of Nepal in August and September, 2011. Species were identified by morphological observation of basidiomata using a stereomicroscope and by genetic analyses of samples. Morphological observations were carried out using Nikon Eclipse 80i stereomicroscope (Nikon, Tokyo). For microscopic observation, pieces of dried fungal material were mounted in 3% (w/v) KOH or Melzer's reagent [32]. Twenty measurements were made per element (spore, basidia, cystidia, and other tissue features) for each specimen.

Genetic analysis was carried out on the internal transcribed spacer region (ITS) of ribosomal DNA. Extraction of genome DNAs from the mushroom samples was performed with ISOPLANT II (NIPPON GENE CO., LTD, Tokyo, Japan) with some modifications. DNA samples were kept at −20°C until used for PCR amplifications. The region between the genes 18S rRNA and 28 S rRNA was amplified using ITS1 and ITS4B [33, 34] primers. A 50 *μ*L reaction mixture was prepared with 1 U Tks Gflex DNA Polymerase (Takara Bio, Inc., Shiga, Japan), 0.5 *μ*M of each primer, 1× Gflex PCR buffer with 1 mM MgCl_2_ and 200 *μ*M dNTPs, and 50 ng of genomic DNA as template according to the manufacturer's instructions. Amplification reaction was performed in a TProfessional Thermocycler (Biometra GmbH, Göttingen, Germany).

An amplified DNA fragment was ligated to the pTA2 plasmid vector (Toyobo, Osaka, Japan). Recombinant plasmid DNA was introduced into *E. coli* DH5*α* competent cells and then isolated with an alkaline lysis method. The nucleotide sequence was determined by Applied Biosystems 3130xl Genetic analyzer (Life Technologies Corporation, Carlsbad, CA, USA). A homology search of the determined nucleotide sequence was carried out using a BLAST server [35].

### 2.2. Mushroom Extract Preparation

Mushroom samples were air-dried and then kept in an air-ventilated oven at 35°C for 10 hours and at 45°C for 1 hour. Twenty-nine samples, after being ground into powder, were extracted in 24 hours at room temperature with reverse osmosis water and ethanol (Wako Pure Chemical Industries, Japan), using an orbital shaker for obtaining 58 extracts. Water extracts were lyophilized, while ethanol extracts were rotary evaporated to dryness when preparing samples for assays. The resultant extracts were kept in glass-capped vials sealed with parafilm and stored in a cool place until assayed.

### 2.3. Phenolic Content Determination

Total phenolic content was determined by a method described by Singleton and Gillespie [36, 37] with some minor modifications. This assay is based on the electrons transferred in alkaline medium from phenolic compounds to blue-colored phosphomolybdic/phosphotungstic acid complexes which have maximum absorbance at 765 nm. Details of the procedures are as follow: 50 *μ*L of sample solution was mixed well with 100 *μ*L of 10% Folin-Ciocalteu solution (a mixture of Na_2_WO_4_, Na_2_MoO_4_, Li_2_SO_4_, HCl, and H_3_PO_4_ with an appropriate ratio) in a 1.5 mL plastic tube. The mixture was equilibrated for several minutes and then 400 *μ*L of 7.5% Na_2_CO_3_ was added to the tube and the reaction mixture was incubated at room temperature for 60–90 minutes. After the incubation period, reaction tubes were centrifuged at 6000 rpm for 2 minutes whenever necessary, 200 *μ*L of supernatant of samples (or blank) was transfer to an optically clear 96-well microplate, and the absorbance was measured at 765 nm using Molecular Devices FlexStation 3 Microplate Reader. Data were managed by SoftMax Pro 5.4.1 software. Gallic acid was used as the standard and was measured in the same conditions as the samples.

### 2.4. Free Radical Scavenging by the ORAC Assay

This assay measures the oxidative degradation of the fluorescence of fluorescein after being mixed with the free radical generator AAPH (2,2′-azobis(2-amidino-propane)dihydrochloride). Heating AAPH is said to produce the peroxyl radical, which damages fluorescein molecules resulting in the loss of fluorescence. Antioxidants suspected to be contained in extracts are considered to protect the fluorescein molecules from this oxidative degeneration. The degree of protection was quantified using a fluorometer. In this study, both water and ethanol extracts were dissolved in 75 *μ*M phosphate buffer (pH 7.4) for use in the ORAC assays, but ethanol extracts were pretreated with a small amount of acetone, final concentration of which in the assay reaction was less than 0.1%. Experiments were conducted in 96-well plates as described previously [38, 39] with some modification, and the main steps were as follows First, 20 *μ*L sample, buffer and trolox solutions were added into the sample, blank, and control wells, respectively. Second, 200 *μ*L fluorescein solution was added into the same wells. After 10 minutes incubation at 37°C, 75 *μ*L of 37°C pre incubated AAPH working solution was also injected into the wells. Finally, fluorescence degradation was measured over 90 minutes, 30 second intervals using Molecular Devices FlexStation 3 Microplate Reader; the excited wavelength and emission wavelengths were 485 nm and 535 nm, respectively. Data were managed by SoftMax Pro 5.4.1. The minimum and maximum concentrations of extracts in buffer were 6.25 and 50 *μ*g/mL, respectively. In the control assay 6.25, 12.5, 25, and 50 *μ*M trolox solutions were used to make the standard curve. All chemicals used for the ORAC assay were of analytical grade and purchased from Wako Chemical, Osaka, Japan.

### 2.5. Free Radical Scavenging by DPPH Radical

The radical scavenging activity of mushroom extracts against the DPPH^•^ radical (2,2-diphenyl-2-picrylhydrazyl hydrate; Sigma-Aldrich, Steinheim, Germany) was determined by the method of Brand Williams modified by Dudonné et al. [40, 41]. DPPH radicals have an absorption maximum at 515 nm; upon reduction by the antioxidant, the solution color fades and the reaction progress is easily monitored by a spectrophotometer (UVmini-1240, Shimadzu, Kyoto, Japan). Determination procedures were as follow: 3 mL of 6 × 10^−5^ M DPPH^•^ solution (prepared daily) was mixed with 100 *μ*L of methanolic solutions of mushroom extracts (maximum dissolved concentration); after 20 min incubation for at 37°C, absorbance decrease of the mixture was monitored at 515 nm (*A*_*s*_). Blank samples with 100 *μ*L of methanol in the above DPPH^•^ solution were prepared and measured daily at same wavelength (*A*_*b*_). The experiment was carried out in triplicate. Radical scavenging activity was calculated using the following formula.

(∗) $$\begin{array}{c} \text{Inhibition}\;\text{rate}\left(\%\right)=\left[\frac{A_{b}-A_{s}}{A_{b}}\right]\times 100. \end{array}$$

### 2.6. Free Radical Scavenging by ABTS Radical

ABTS assay was mostly based on the methods described previously [42] in which ABTS^•+^, the oxidant, was generated by persulfate oxidation 2,2′-azinobis(3-ethylbenzothiazoline-6-sulfonic acid). Specifically, to 5 mL of 7 mM ABTS ammonium aqueous solution, 88 *μ*L of 140 mM potassium peroxydisulfate (K_2_S_2_O_8_) was added and the resulting mixture was then allowed to stand at room temperature for 12–16 hour to yield a dark blue solution. The mixture was then adjusted by 99.5% ethanol so that it gave an absorbance of 0.7 ± 0.02 units at 734 nm for a making working solution. One milliliter of working solution was mixed with 10 *μ*L of mushrooms extract (maximum dissolved concentration) and shaken well for 10 seconds; after 4 minutes of incubation at 30°C, the absorbance of the reaction mixture was measured at 734 nm (UVmini-1240, Shimadzu, Kyoto, Japan) to give “*A*_*s*_” values. Ethanol 99.5% was used as a blank (absorbance was “*A*_*b*_”) and the inhibition rates were calculated using ((∗)).

### 2.7. Detection of Intracellular UVB-Induced H_2_O_2_

Intracellular H_2_O_2_ was assessed using immortal human keratinocyte line (HaCaT) as cell model. HaCaT cells (Cell Line Service, Eppelheim, Germany) were cultured in DMEM supplemented with L-glutamine, 10% fetal bovine serum and 1% penicillin/streptomycin antibiotic solution. After being cultured for two days at 37°C in a 95% air/5% CO_2_ atmosphere, cells were removed from culture dish by trypsinization and seeded at a density of 4 × 10^5^ cells/dish in a 5 cm petri dish. After two days culturing, medium was removed and cells were exposed to 10 mJ/cm^2^ UVB (CL-1000 Ultraviolet Crosslinker, UVP, Upland, CA, USA). Soon after UVB irradiation, cells were refilled with cultured medium supplemented with 10 ppm of mushroom extracts. After one more day incubation, cells were transferred to 96-well *μ*Clear Fluorescence Black Plate (number 655090, Greiner Bio-one, Tokyo, Japan) at a density of 2 × 10^4^ cells/well and incubated for 24 hours. Nucleus was stained by Hoechst 33342 (Dojindo, Kumamoto, Japan) and the amount of intracellular H_2_O_2_ was quantified based on the amount of difluorofluorescein (DFF) released from the reaction of H_2_O_2_ and BES-H_2_O_2_-Ac (Wako Chemical, Osaka, Japan). The images of each well were acquired from IN Cell Analyzer 1000 (GE Healthcare, Amersham Place, UK) using 360 nm (Hoechst 33342) and 480 nm (BES-H_2_O_2_-Ac) excitation filters and monitored through 460 nm and 535 nm emission filters, respectively. The images of Hoechst 33342 and BES-H_2_O_2_-Ac staining were analyzed using Developer software and resulted data were then applied to Spotfire Decision Site Client 8.2 software for visualizing the results. Cells unexposed to UVB irradiation were used as controls; cells exposed to UVB and cultured in the presence or absence of resveratrol (10 ppm of final concentration) were used as positive or negative control, respectively.

### 2.8. Angiotensin-Converting Enzyme Inhibitory Assay

Water extracts were dissolved in milli-Q water (Millipore, MA, USA) and those that were difficult to dissolve in water were pretreated with a small amount of ethanol before being dissolved in milli-Q water (final concentration of organic solvent in enzyme reactions was less than 1%). Both types of extract were subjected to ACE-inhibitory assay using Dojindo ACE Kit-WST test kit (Dojindo Laboratories, Kumamoto, Japan). Details of the method's principle can be found elsewhere [43]. Briefly, the enzymatic reaction was initiated by the ACE and aminoacylase in the mixture containing 3HB-GGG (3-hydroxybutyrate glycylglycylglycine) and the ACE-inhibitor. The mixture was then incubated at 37°C for 60 min. During this incubation, the substrate, 3HB-GGG, was enzymatically cut into 3HB-G and G-G and then 3HB and G. The yield of 3HB was monitored indirectly through formazan concentration, which was measured at 450 nm after 10 minute reaction at 25°C.

Testing procedures were run according to the manufacturer's instructions using a 96-well plate without modification, and the inhibition rate was calculated based on a comparison of the optical absorbance of samples-treated wells (*A*_*s*_), control wells (*A*_*c*_), and blank wells (*A*_*b*_). Absorbance was measured at 450 nm using the microplate reader Biotek-ELX800 (BioTek, Vermont, USA). Inhibition rates were calculated using the following equation.

(1) $$\begin{array}{c} \text{Inhibition}\;\text{rate}\;\left(\%\right)=\left[\frac{A_{c}-A_{s}}{A_{c}-A_{b}}\right]\times 100. \end{array}$$

Samples were suspected to inhibit the ACE activity, and therefore inhibit the formation of formazan. The more strongly inhibitory the activity of the samples, the less color appeared in the final solution.

### 2.9. Statistical Analysis

Each ORAC experiment was repeated four times, while the ACE-inhibitory assay and phenolic content determination were performed in triplicate. The results are expressed as mean ± SD. The correlation coefficient between phenolic content and antioxidant assays was determined by least-square linear regression analysis using Microsoft Excel 2007.

## 3. Results and Discussion

### 3.1. Mushroom Collection and Identification

As shown in Table 1, 29 mushroom samples, collected from the mountainous area of Nepal were first identified by morphological observation. Some mushrooms for which ITSs were obtained were subjected to a BLAST search via INSDC.

Mushroom samples were collected at mass from 11.6 to 117.7 g in dried weight. Purified genome DNAs were successfully obtained from the mushroom samples. DNA fragments containing the ITS sequence were amplified in the 705 to 894 bp range. From BLAST search results, the mushroom listed in Table 1 were identified. Six samples (from N001 to N006) were included in the genus *Ganoderma*, and three samples (N016, N018, and N019) belonged to the genus *Phellinus*. Samples N009, N011, N014, N027, and N028 were identified as *Trametes versicolor*.

### 3.2. Antioxidant Activities

#### 3.2.1. Free Radical Scavenging by the ORAC Assay

Many methods have been developed for measuring antioxidant capacity *in vitro*. The underlying chemistry, advantages, and disadvantages have also been well documented and reviewed [44, 45]. Among these methods, the oxygen radical absorbance capacity (ORAC) method, with some modifications that have been made over time, has been widely used to evaluate the antioxidant activity of many herbal extracts, food additives, and even biological samples [46]. The existence of the (USDA) US Department of Agriculture ORAC database and the recently launched web-based database for this index [47] show the scientific community's estimation of the ORAC assay for measuring antioxidant capacity of herbal samples. In the initial checking for antioxidant activity of mushroom extracts in this study, we ran ORAC experiments in which fluorescein was used as fluorescent probe in a 96-well plate assay as described above.

The results of ORAC assays of samples are shown in Table 2. ORAC values (*μ*mol TE/g extract) ranged from 342.8 to 21015.4 for ethanol extracts and from 83.2 to 1196.9 for water extracts. Among the samples *Inonotus andersonii* and *Phellinus gilvus* ethanol extracts showed extremely high activity. This is the first time such high ORAC values have been seen for mushroom extracts. Until now, such high ORAC values have only been reported for extracts of well-known antioxidant spices like cloves, pimento, and cinnamon [41, 48]. High antioxidant activities have also been reported for some mushrooms in *Inonotus* species such as *I. hispidus* and *I. obliquus *[49, 50], but we could not find any published report on the antioxidant capacity of the *I. andersonii* mushroom. It is worth noting here that the main antioxidative compounds isolated from above-mentioned *Inonotus* mushrooms are hispidin and hispidin moiety-contained compounds such as inonotusin A and B in *I. hispidus* [49], inonoblins, and phelligridins in *I. obliquus *[51]. From these results, we think that *I. andersonii *may also contain such compounds and this mushroom should be a good candidate for future antioxidant researches.

#### 3.2.2. Phenolic Content, ABTS, and DPPH Radical Scavenging Assays

Phenolic compounds are considered one of the major groups of nonessential dietary components which have been suggested to be beneficial for human health and their physiological importance is said to relate to their abilities to chelate metals, inhibit lipoxygenase, and scavenge free radicals [52]. The Folin-Ciocalteu method is often used to estimate the phenolic content of plant extract samples although the reagent used for determining phenolic content does not react exclusively with phenolics and has even been proven to be affected by a variety of compounds such as thiol derivatives, vitamin derivatives, amino acids, and metal complexes [53]. Thus, the reagent often overestimates the phenolic contents in samples, but because it is a cheap, simple, convenient and, in some aspects, useful method for determining total phenolic content, Folin-Ciocalteu is still widely used to estimate the total antioxidant capacity of samples. Many studies on spices, vegetables, fruits, and plants extracts have shown a good relationship between phenolic content and antioxidant activity [41, 53–55]. It is also generally accepted that the main antioxidants in mushrooms are phenolics, mainly phenolic acids [56]. To confirm the relationship of phenolic compounds in mushrooms and their antioxidant activities we selected 10 mushroom samples which had the highest ORAC values and carried out experiments for determining phenolic content and other radical scavenging activities. The correlation coefficients between phenolic content and antioxidant activity resulting from different assays were also calculated and the results were shown in Table 3.

Experimental results showed good relationships between phenolic content and antioxidant activities in which the correlation coefficient *R* of phenolics and ORAC, ABTS, and DPPH activities were 0.923, 0.936, and 0.986, respectively. The close correlation between phenolic content and ABTS inhibition is not surprising since the methods used to determine phenolics and ABTS inhibition rates are both based on the electron transfer ability of the sample's components. However, while the ORAC assay is based on hydrogen atom transfer reactions, we still could see a good correlation between phenolic content and ORAC values. This may come from the fact that phenolic compounds are not only a rich electron source, but the phenolic hydroxy group can also act as a hydrogen donor supplying hydrogen atom to wipe out peroxy radicals by forming stabilized phenoxyl radicals in the ORAC assay. In this context, phenolic compounds can be both electron and hydrogen atom donors, and therefore can be in good correlation with both the ORAC and ABTS assays. The very high correlation found between DPPH assays and total phenolic content with *R* equal to 0.986 indicates a close relationship between phenolic compound concentration in mushroom extracts and their nitrogen-radical scavenging capacities.

Despite the fact that there have been many studies referring to the antioxidant activity of mushroom, almost no report has mentioned a correlation between mushroom genus (or family) and antioxidant activity. Our present results suggest such a relationship. For example, all studied *Ganoderma* (Ganodermataceae) samples had a medium antioxidant activity, while *Phellinus* and *Inonotus* samples (Hymenochaetaceae) showed quite high activity. Some previous discrete studies [51, 57–61] have also shown the high antioxidant capacity of many mushrooms in the *Inonotus* and *Phellinus *genera. This consistency across different studies results can be used to consolidate and direct future research on antioxidant activity. Assuming that this genus bioactivity relationship can be further established, we think that these genera could be good candidates for studies of mushroom's antioxidants properties in the future.

#### 3.2.3. Intracellular UVB-Induced H_2_O_2_

Ultraviolet-induced ROS cause chemical modification and oxidative stress and play an important role in photoaging. After being created, ROS activate many cell surface cytokines and growth factor receptors which stimulate transcriptions of matrix metalloproteinases that significantly contribute to the skin aging process [62, 63]. Many antioxidants such as vitamin C, vitamin E, carotenoids, and, especially, polyphenols have been reported with ability of enhancing resistance to oxidative stress and preventing skin aging [12, 14, 15, 17]. In this study 10 mushroom's extracts having highest antioxidant activity (highest phenolic contents) were subjected to the UVB-induced H_2_O_2_ generation assay to check the ability of samples on antioxidative stress effects using HaCaT cells as cellular model. The results were shown in Table 4.

Four among ten selected samples showed good protection effect against UVB-induced H_2_O_2_ generation. The levels of intracellular H_2_O_2_ in cells treated with these samples were as low as in control. Two highest phenolics-containing samples *I. andersonii* and *P. gilvus* also belonged to group of these 4 samples. Considerably, cells treated with *P. gilvus* even showed lower level of H_2_O_2_ than control's implying the ability of this sample on scavenging intracellular generated H_2_O_2_ or other types of ROS. In previous parts, we proposed the use of this mushroom for further investigation on antioxidant activity and this result confirmed our proposal about targeting the mushroom as a potent candidate for future studies as antioxidative stress agents.

Surprisingly, resveratrol not only failed to show protection effects against UVB-induced H_2_O_2_ but also performed a stimulation of generation of this compound. This result was different from previous reports of Park [64] on the protective activity of resveratrol against H_2_O_2_-induced oxidative stress. However, while Park incubated HaCaT cells with resveratrol before applying UVB, in this study we applied UVB before treating cells with resveratrol. UVB-exposure has been reported to decrease the catalase activity [65], while pretreatment of cells with resveratrol has been reported to increase the expression of SOD and glutathione peroxidase [66] and catalase [67], major enzymes responsible for the inactivation of ROS. The differences of our results and previous report's could result from the difference in resveratrol-treatment methods.

#### 3.2.4. ACE-Inhibitory Assay

Water and ethanol extracts of 29 mushrooms samples were used for screening the ACE-inhibitory effect using Dojindo ACE test kits. Each test was repeated three times and inhibition rates were calculated based on a comparison of blank and control samples. Results are shown in Table 5.

High blood pressure is one of the major independent risk factors for cardiovascular diseases and is considered a worldwide health problem. Angiotensin-I-converting enzyme (EC 3.4.15.1; ACE) plays a crucial role in blood-pressure regulation by converting angiotensin I to angiotensin II, a potent vasoconstrictor. Therefore, the inhibition of ACE activity is a major target in the prevention of hypertension [68]. Until now, ACE-inhibitors have been mainly sourced from food protein, especially milk protein. Many milk protein-derived peptides have demonstrated inhibitory effects on ACE *in vitro *[69–71] and on antihypertension *in vivo *[72–74].

Recently, mushrooms have also been considered as good candidate sources of hypotensive agents. Several peptides and proteins extracted from mushrooms have been shown to have an ACE-inhibitory effect. Many mushroom extracts have been screened for this activity [26, 75–77], and most of the time, the dominant ACE-inhibition extracts have been aqueous. Consistently with previous results, our study also showed a predominance of water extracts for ACE-inhibition. While there were 15 water extracts which showed rather high inhibitory effect with inhibition rates higher than 50% at 100 *μ*g/mL, only one ethanol extract showed more than 50% inhibition at this concentration. Besides traditionally well-known mushrooms such as *Ganoderma lingzhi* and *Trametes versicolor*, other nonmedicinal mushrooms like *Phlebia tremellosa* and *Heterobasidion* sp. samples also showed high inhibition activity. According to Lindequist et al. [4], the responsible bioactive compounds in mushrooms belong to several chemical groups; usually they are polysaccharides, triterpenoids, and proteins. As mentioned above, several ACE-inhibitory peptides and proteins have been identified from mushroom water extracts. From ethanol or methanol extracts only some ganoderic acids [23] and nicotianamine [76] with ACE-inhibitory capacity have been identified. Recent studies indicated that phenolic compounds can also play a role in the inhibition of ACE [77, 78]. In this study, the average inhibition against ACE of *I. andersonii* and *P. gilvus* ethanol extracts (the two highest-phenolic-content samples) could be explained by the action of phenolic compounds in the mushroom extracts.

Among the studied samples, it seems that *Phlebia tremellosa* contained potent compounds having high ACE-inhibition capacity. To confirm the potential of mushrooms for ACE-inhibitory activity, we performed the IC_50_ value determination for water extracts of this mushroom and compared with *Ganoderma lingzhi's *capacity. The IC_50_ of *Phlebia tremellosa* was 16 times higher than that of* Ganoderma lingzhi* sample, with values of 32 *μ*g/mL and 2 *μ*g/mL, respectively. These results confirmed the potential of this mushroom for ACE-inhibition, and it should be pursued in future studies.

Besides the fact that the ACE-inhibitory capacity of most mushrooms (except for *Ganoderma lingzhi*) in this study has never been reported, our results also indicated a clear relationship between mushroom genus and certain activities, as mentioned above. Five of six *Ganoderma* and four of five *Trametes* mushroom samples showed high inhibition rates at the studied concentration. This correlation may result from the similarity of the chemical structures of metabolites provided by fungal species belonging to the same genus [77]. Further investigation and more samples are needed to confirm the speculation, but assuming such correlations can be established, this should be valuable information for directing future researches.

## 4. Conclusion

Twenty-nine mushroom samples of 21 species in 14 genera collected in Nepal were checked for antioxidant and angiotensin-converting enzyme *in vitro* inhibition capacity. Beside *Phellinus gilvus* which was reported as a potent mushroom for isolating antioxidant compounds in some previous studies, this time we showed that *Inonotus andersonii* is also a promising candidate for antioxidant investigation with an antioxidant capacity equivalent to the well-known antioxidant spice, cloves. The H_2_O_2_-scavenging assay on HaCaT cells also revealed the potential of these mushrooms in the prevention of oxidative stress. From the fact that other samples of the *Phellinus* genus also showed high antioxidant activity, we deduced the potential of this genus as an important antioxidant source for future studies. ACE-inhibition assays indicated that *Phlebia tremellosa* is a novel and potent candidate for antihypertensive studies. This mushroom exhibited even higher *in vitro* ACE-inhibition activity than *Ganoderma lingzhi*, with the IC_50_ values of the two mushrooms at 32 *μ*g/mL and 2 *μ*g/mL, respectively. With half of the mushrooms samples herein being reported for antioxidant properties for the first time and most of the mushrooms having never been reported for ACE-inhibitory activity, information from this study should be a valuable reference for future studies on antioxidant and ACE-inhibitory activities of mushrooms.

## Figures and Tables

**Table 1:** Information related to mushrooms used in present study.

Number	Scientific name	Locus*	Habitat	INSDC Acc. number
N001	*Ganoderma carnosum *	Mt. Phulchoki/2765 m	Decayed wood	AB763348
N002	*Ganoderma lingzhi *	Mt. Phulchoki/2765 m	Decayed wood	AB811848
N003	*Ganoderma australe *	Mustang/3150 m	Decayed wood	AB811849
N004	*Ganoderma australe *	Mt. Phulchoki/2765 m	Decayed wood	AB811850
N005	*Ganoderma australe *	Mt. Phulchoki/2765 m	Decayed wood	Not determined
N006	*Ganoderma australe *	Dawachok/1500 m	Decayed wood	AB811852
N007	*Postia stiptica *	Dawachok/1500 m	Decayed wood	AB811853
N008	*Phlebia tremellosa *	Mt. Phulchoki/2765 m	Soil	AB811854
N009	*Trametes versicolor *	Mt. Phulchoki/2765 m	Decayed wood	AB811855
N010	*Inonotus andersonii *	Mt. Phulchoki/2765 m	Soil	AB811856
N011	*Trametes versicolor *	Mt. Phulchoki/2765 m	Decayed wood	AB811857
N012	*Inonotus *sp. 1	Mt. Phulchoki/2765 m	Living tree	Not determined
N013	*Heterobasidion linzhiense *	Surya Binayak/1400	Living tree	AB811859
N014	*Trametes versicolor *	Mt. Phulchoki/2765 m	Living tree	AB811860
N015	*Heterobasidion linzhiense *	Mt. Phulchoki/2765 m	Living tree	AB811861
N016	*Phellinus gilvus *	Mt. Phulchoki/2765 m	Decayed wood	AB811862
N017	*Inonotus *sp. 2	Mt. Phulchoki/2765 m	Decayed wood	Not determined
N018	*Phellinus conchatus *	Nagarkot/2500 m	Decayed wood	AB811863
N019	*Phellinus conchatus *	Nagarkot/2500 m	Decayed wood	AB811864
N020	*Inocybe *sp.	Mt. Phulchoki/2765 m	Soil	Not determined
N021	*Collybia peronata *	Nagarkot/2500 m	Fallen leaves	Not determined
N022	*Inonotus *sp. 3	Mt. Phulchoki/2765 m	Decayed wood	AB811865
N023	*Lactarius hatsudake *	Mustang/3150 m	Soil	Not determined
N024	*Lenzites betulina *	Mt. Phulchoki/2765 m	Soil	AB811866
N025	*Panellus *sp.	Mt. Phulchoki/2765 m	Decayed wood	Not determined
N026	*Rigidoporus* sp.	Surya Binayak/1400	Decayed wood	Not determined
N027	*Trametes versicolor *	Mt. Phulchoki/2765 m	Decayed branch**	AB811867
N028	*Trametes versicolor *	Mt. Phulchoki/2765 m	Decayed branch**	AB811868
N029	*Tricholoma caligatum *	Mt. Phulchoki/2765 m	Soil	Not determined

*Number in “m” is average height of samples-collection area; **Decay branch of living tree.

**Table 2:** ORAC values (*μ*mol TE/g extract) of mushrooms extracts.

Number	Scientific name	ORAC values	
		EtOH	H_2_O
N001	*Ganoderma carnosum *	1938.6 ± 64.5	764.2 ± 29.8
N002	*Ganoderma lingzhi *	2136.2 ± 100.8	1046.1 ± 18.5
N003	*Ganoderma australe *	1406.9 ± 71.4	663.0 ± 19.4
N004	*Ganoderma australe *	1602.9 ± 88.5	497.1 ± 69.7
N005	*Ganoderma australe *	1781.4 ± 123.3	813.5 ± 23.6
N006	*Ganoderma australe *	2578.5 ± 99.0	1196.9 ± 48.7
N007	*Postia stiptica*.	449.5 ± 27.7	660.5 ± 15.5
N008	*Phlebia tremellosa *	960.1 ± 38.1	629.2 ± 14.6
N009	*Trametes versicolor *	615.6 ± 34.0	650.0 ± 6.4
N010	*Inonotus andersonii *	21015.4 ± 121.3	83.2 ± 31.9
N011	*Trametes versicolor *	2168.0 ± 33.1	111.3 ± 36.7
N012	*Inonotus *sp. 1	1848.0 ± 77.6	761.1 ± 15.4
N013	*Heterobasidion linzhiense *	616.1 ± 83.1	655.9 ± 26.8
N014	*Trametes versicolor *	410.6 ± 49.8	745.3 ± 3.7
N015	*Heterobasidion linzhiense *	691.0 ± 22.1	419.7 ± 19.8
N016	*Phellinus gilvus *	9564.0 ± 281.5	280.7 ± 22.4
N017	*Inonotus *sp. 2	1746.6 ± 100.9	358.2 ± 43.0
N018	*Phellinus conchatus *	3856.4 ± 296.6	558.1 ± 18.9
N019	*Phellinus conchatus *	4431.5 ± 211.6	570.3 ± 22.4
N020	*Inocybe *sp.	472.7 ± 38.8	447.6 ± 35.8
N021	*Collybia peronata *	802.4 ± 45.9	461.3 ± 15.9
N022	*Inonotus *sp. 3	342.8 ± 7.9	936.5 ± 69.5
N023	*Lactarius hatsudake *	425.5 ± 27.4	941.9 ± 54.9
N024	*Lenzites betulina *	548.9 ± 14.3	741.6 ± 13.8
N025	*Panellus *sp.	462.8 ± 12.0	1070.1 ± 42.1
N026	*Rigidoporus* sp.	1025.9 ± 48.7	534.1 ± 28.9
N027	*Trametes versicolor *	435.3 ± 14.2	984.4 ± 14.1
N028	*Trametes versicolor *	522.2 ± 18.8	499.9 ± 26.5
N029	*Tricholoma caligatum *	620.5 ± 18.8	247.1 ± 10.8

The bold values show the samples with high ORAC value.

**Table 3:** Phenolic content, ORAC values, ABTS, and DPPH radical scavenging results of top 10 extracts.

Number	Scientific name	Ethanol extract			
		Total phenolic (mg GAE/g)	ORAC	ABTS (%)	DPPH (%)
N010	*Inonotus andersonii *	310.8 ± 2.7	21015.4 ± 121.3	36.4 ± 1.0 (63.5)	72.9 ± 2.1 (102.0)
N016	*Phellinus gilvus *	258.7 ± 4.3	9564.0 ± 281.5	38.1 ± 0.4 (56.0)	55.2 ± 3.2 (109.0)
N017	*Inonotus *sp. 2	97.1 ± 0.9	1746.6 ± 100.9	24.1 ± 0.3 (62.5)	22.4 ± 1.6 (106.0)
N006	*Ganoderma australe *	88.3 ± 2.4	2578.5 ± 099.0	21.5 ± 0.7 (63.5)	19.2 ± 0.7 (108.0)
N005	*Ganoderma australe *	82.7 ± 8.2	1781.4 ± 123.3	16.1 ± 2.3 (53.5)	14.7 ± 0.9 (100.0)
N019	*Phellinus conchatus *	74.9 ± 1.3	4431.5 ± 211.6	19.3 ± 0.2 (58.5)	14.5 ± 1.5 (103.0)
N018	*Phellinus conchatus *	68.2 ± 0.2	3856.4 ± 296.6	12.6 ± 0.3 (47.5)	17.0 ± 0.2 (115.0)
N001	*Ganoderma carnosum *	70.2 ± 3.0	1938.6 ± 064.5	16.0 ± 2.4 (61.0)	11.3 ± 1.3 (103.0)
N002	*Ganoderma lingzhi *	66.8 ± 3.0	2136.2 ± 100.8	15.1 ± 0.4 (63.0)	10.7 ± 0.6 (126.0)
N011	*Trametes versicolor *	50.4 ± 0.5	2168.0 ± 033.1	10.3 ± 0.3 (74.0)	NA**

Correlation coefficient*		—	**R** = 0.923	**R** = 0.936	**R** = 0.986

*Correlation coefficients in each column were between phenolic content and correspondent antioxidant activity; **not available; numbers in bracket of ABTS and DPPH columns were final concentrations (*μ*g/mL).

The bold values show high correlation coefficient.

**Table 4:** Effect of mushrooms extract on UVB-induced intracellular H_2_O_2_ generation in HaCaT cells.

Number	Scientific name	DFF*	Number	Scientific name	DFF*
N016	*Phellinus gilvus *	7.3 ± 6.1	N019	*Phellinus conchatus *	31.7 ± 3.1
N001	*Ganoderma carnosum *	19.0 ± 9.6	N018	*Phellinus conchatus *	32.7 ± 4.0
N010	*Inonotus andersonii *	26.7 ± 4.0	N002	*Ganoderma lingzhi *	35.3 ± 7.4
N005	*Ganoderma australe *	26.7 ± 7.6	N011	*Trametes versicolor *	35.7 ± 4.9
N017	*Inonotus *sp. 2	31.3 ± 10.7	N006	*Ganoderma australe *	53.3 ± 5.8

Control		26.3 ± 7.0	Resveratrol		53.3 ± 4.0
Negative control		36.0 ± 9.8		

*DFF: difluorofluorescein fluorescent intensity; proportionally related to H_2_O_2_ concentration.

The bold values show low difluorofluorescein fluorescent intensity compared to control and negative control.

**Table 5:** ACE-inhibition rate of mushroom extracts at concentration of 100 µg/mL.

Number	Scientific name	ACE inhibition (%)	
		EtOH	H_2_O
N001	*Ganoderma carnosum *	22.99 ± 6.45	71.30 ± 2.22
N002	Ganoderma lingzhi*	21.42 ± 3.77	76.98 ± 1.22
N003	*Ganoderma australe *	22.11 ± 5.03	38.61 ± 4.65
N004	*Ganoderma australe *	15.37 ± 7.67	50.72 ± 6.43
N005	*Ganoderma australe *	20.99 ± 3.44	65.26 ± 6.76
N006	*Ganoderma australe *	33.10 ± 8.53	61.59 ± 2.98
N007	*Postia stiptica *	18.23 ± 1.16	26.81 ± 6.71
N008	Phlebia tremellosa**	6.14 ± 1.19	92.57 ± 1.25
N009	*Trametes versicolor *	19.72 ± 2.11	38.18 ± 0.41
N010	*Inonotus andersonii *	52.76 ± 1.80	39.38 ± 7.52
N011	*Trametes versicolor *	17.03 ± 0.82	58.92 ± 7.82
N012	*Inonotus *sp. 1	18.25 ± 2.47	18.22 ± 1.40
N013	*Heterobasidion linzhiense *	1.47 ± 3.33	54.97 ± 2.67
N014	*Trametes versicolor *	nd***	69.09 ± 1.41
N015	*Heterobasidion linzhiense *	nd	73.38 ± 3.08
N016	*Phellinus gilvus *	40.96 ± 2.60	13.80 ± 4.16
N017	*Inonotus *sp. 2	14.54 ± 4.45	nd
N018	*Phellinus conchatus *	18.21 ± 0.86	nd
N019	*Phellinus conchatus *	19.56 ± 4.59	48.39 ± 4.00
N020	*Inocybe *sp.	nd	56.05 ± 7.40
N021	*Collybia peronata *	9.72 ± 1.23	38.99 ± 7.34
N022	*Inonotus *sp. 3	nd	15.71 ± 1.71
N023	*Lactarius hatsudake *	nd	nd
N024	*Lenzites betulina *	16.31 ± 1.03	84.87 ± 2.04
N025	*Panellus *sp.	23.92 ± 1.49	35.36 ± 3.32
N026	*Rigidoporus* sp.	1.95 ± 2.32	10.17 ± 9.07
N027	*Trametes versicolor *	4.22 ± 5.10	71.10 ± 1.28
N028	*Trametes versicolor *	0.17 ± 3.17	75.40 ± 1.80
N029	*Tricholoma caligatum *	nd	55.40 ± 3.89

*IC_50_ = 32 µg/mL; **IC_50_ = 2 µg/mL; ***nd: not detected.

The bold values show high ACE inhibition.
